# Modulation of Chemokine Responses: Synergy and Cooperativity

**DOI:** 10.3389/fimmu.2016.00183

**Published:** 2016-05-19

**Authors:** Amanda E. I. Proudfoot, Mariagrazia Uguccioni

**Affiliations:** ^1^Novimmune S.A., Geneva, Switzerland; ^2^Laboratory of Chemokines in Immunity, Institute for Research in Biomedicine, Università della Svizzera italiana, Bellinzona, Switzerland

**Keywords:** chemokines, cell migration, synergy, oligomerization, glycosaminoglycan

## Abstract

Chemokine biology is mediated by more complex interactions than simple monomolecular ligand–receptor interactions, as chemokines can form higher order quaternary structures, which can also be formed after binding to glycosaminoglycans (GAGs) on endothelial cells, and their receptors are found as dimers and/or oligomers at the cell surface. Due to the complexity of the chemokine binding and signaling system, several mechanisms have been proposed to provide an explanation for the synergy observed between chemokines in leukocyte migration. Pioneering studies on interactions between different chemokines have revealed that they can act as antagonists, or synergize with other chemokines. The synergism can occur at different levels, involving either two chemokine receptors triggered simultaneously or sequentially exposed to their agonists, or the activation of one type of chemokine receptor triggered by chemokine heterocomplexes. In addition to the several chemokines that, by forming a heterocomplex with chemokine receptor agonists, act as enhancers of molecules of the same family, we have recently identified HMGB1, an endogenous damage-associated molecular patterns (DAMPs) molecule, as an enhancer of the activity of CXCL12. It is now evident that synergism between chemokines is crucial at the very early stage of inflammation. In addition, the low-affinity interaction with GAGs has recently been shown to induce cooperativity allowing synergy or inhibition of activity by displacement of other ligands.

## Chemokines and Their Receptors

Chemokines are key regulators of leukocyte migration and function, playing fundamental roles both in physiological and pathological immune responses, such as inflammatory diseases (1). The chemokine system includes ~50 ligands, which engage a panel of over 20 chemokine receptors in a promiscuous fashion, which are differentially expressed by all leukocytes and many non-hematopoietic cells (2). Proper tissue distribution of distinct leukocyte subsets, under normal and pathological conditions, is guaranteed by the resulting combinatorial diversity in cell responsiveness to chemokines.

To mediate their activity chemokines bind to cell surface receptors which belong to the largest branch of the γ subfamily of rhodopsin-like G protein-coupled receptors (GPCRs) (3), a receptor superfamily which represents the most successful target of small molecule inhibitors for treating diseases affecting different systems in modern pharmacology. All chemokine receptors couple to heterotrimeric Gα_i_-proteins and accordingly most responses can be fully inhibited by treatment of cells with *Bordetella pertussis* toxin. Today, a total of 19 signaling receptors have been identified: 7 CXCRs (CXCR1–6 and CXCR8), 10 CCRs (CCR1–10), CX3R3, and CKR1. In addition, there are four “atypical” receptors that use alternative signaling pathways, and act mainly by sequestering and degrading the chemokines present in the microenvironment (4). Thus, the ~50 chemokines outnumber their receptors indicating that a receptor can bind more than one chemokine. In addition, several chemokines can also bind to multiple receptors (2, 5). Novel findings indicate that polysialylation of CCR7, the central chemokine receptor controlling immune cell trafficking to secondary lymphatic organs, is essential for the recognition of the CCR7 ligand CCL21 (6), and that the glycosylation pattern of this receptor shapes receptor signaling (7), suggesting that this further level of control might be shared with other chemokine receptors.

## Chemokine Synergy and Cooperation

A vast range of *in situ* experiments, aimed at understanding which chemokines are produced under specific circumstances, has revealed that a variety of chemokines can be concomitantly produced at the target sites of leukocyte trafficking and homing (8–12). This renders the chemokine system a good target for therapy and has promoted the search by pharmaceutical companies for small molecule chemokine antagonists (13–16). While we understand the effects of different chemokines singly, much less is known about the potential consequences of the concomitant expression of multiple chemokines and their interaction with other inflammatory molecules (17, 18).

The suggestion that chemokines might have additional regulatory mechanisms started with the identification of natural chemokine antagonists. Many reports have demonstrated that certain chemokines can also antagonize non-cognate chemokine receptors, by altering agonist-induced signaling and abrogating cellular responses *via* several mechanisms, including occupancy of the chemokine receptor-binding pocket or signaling through Rac-2 (19–24).

**Figure 1:**
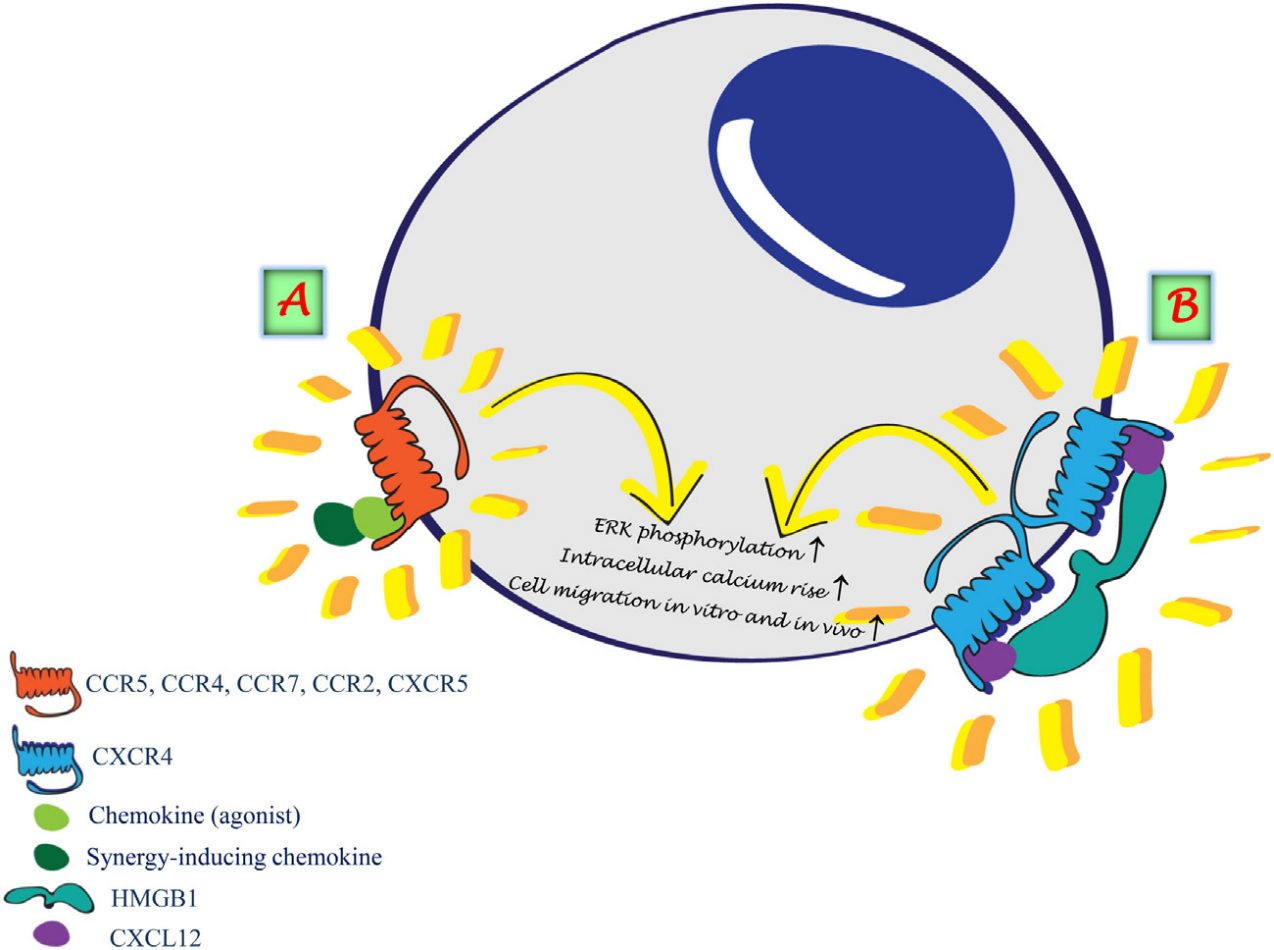
**Synergism induced by the formation of heterocomplexes**. **(A)** Heterocomplex formed between two chemokines renders the agonist more potent on the selective receptor. **(B)** HMGB1 forms a heterocomplex with CXCL12 enhancing CXCL12 potency on CXCR4.

The studies on possible regulatory mechanisms continued when three reports showed that chemokines can synergize to enhance leukocyte functions in response to chemoattractants. The first described a bovine chemokine, regakine 1 that induces enhanced neutrophil migration when combined with CXCL7, CXCL8, and C5a. The receptor or the mechanism of regakine-1-induced synergism is not known. Competition with labeled C5a for binding to neutrophils or receptor-transfected cell lines demonstrated that regakine 1 does not alter receptor recognition. The protein kinase inhibitors 2′ amino 3′ methoxyflavone (PD98059), wortmannin, and staurosporine had no effect on the synergy between C5a and regakine 1 (25). The second study showed that migration of natural IFN-producing cells, a subpopulation of murine and human lymphocytes, to the CXCR3 agonists requires stimulation of CXCR4 by CXCL12. The mechanism by which CXCL12 induces enhanced migration in response to CXCR3 agonists is yet unknown. CXCL12 does not upregulate the expression of CXCR3 and does not increase the affinity of CXCR3 for its agonists (26). The third report (27) showed the same enhanced migration, on human plasmacytoid dendritic cells, in response to CXCR3 agonists induced by stimulation with CXCL12 as observed by Krug et al. (26). These reports undoubtedly indicate, as for the natural antagonist chemokines, that it is necessary to carefully analyze the effects that the concomitant expression of chemokines can have on cell functions and to elucidate the molecular mechanisms governing cell activities at sites of inflammation. Synergism can thus occur at different levels, involving either two chemokine receptors triggered simultaneously or sequentially exposed to their agonists (26–30). We have identified a further mechanism by which chemokines, forming chemokine heteromeric complexes, can activate one type of chemokine receptor (Figure 1A) (31): (i) CXCL13 enhances CCL19 and CCL21 triggering of CCR7 (32); (ii) CXCL10 enhances CCL22 triggering of CCR4 (33); (iii) CCL19 and CCL21 enhance the activity of CCR2 ligands and protect them from degradation (34); and (iv) CXCL9 enhances migration induced by CXCL12 on CXCR4^+^/CXCR3^−^ malignant B cells (35). Other groups have also shown that the synergism between a chemokine agonist and a non-ligand chemokine can enhance the activity of selective chemokine receptors (36–40).

Chemokines have a second important interaction with cell surface expressed glycosaminoglycans (GAGs), which mediates their immobilization on the endothelial surface in order to provide their directional signal (41–43). This interaction was shown to be essential for their ability to recruit cells *in vivo* by the loss of activity of chemokine variants, which had abrogated GAG-binding capacity (44). Without the interaction with endothelial GAGs, most chemokines would be washed away from the local production site, especially under flow conditions, diluted to a concentration below the threshold required for binding, and distributed uniformly throughout the vasculature such that no localized chemotactic signal is generated for leukocytes to allow directional mobilization. Furthermore, differential binding to GAGs plays an important role in localization. Neutrophil recruitment to the lung is greater in response to chemokines that bind GAGs less strongly. This was demonstrated both by mutants of CXCL8 with abrogated GAG binding as well as comparison of another neutrophil chemoattractant, CXCL1. Although increased recruitment was postulated to be mediated by the stronger GAG binder, lower binding capacity resulted in enhanced recruitment, demonstrating that the tissue microenvironment plays a pivotal role in the spatial formation of chemokine gradients and defining GAGs functions (45, 46).

Recently, binding to cell surface GAGs has identified more subtle roles in chemokine biology, where competitive binding of chemokines to GAGs can either induce cooperative enhancement of activity or inhibition of activity by displacement of certain chemokines. Cooperative enhancement has been demonstrated for both classical receptors as well as atypical or non-signaling receptors such as CCX-CKR/ACKR4 (47). In both cases, competitive displacement of the chemokines from GAGs was shown to be responsible for the effects, using modified chemokines lacking the GAG-binding sequence. The competitive displacement is limited to chemokines which bind GAGs strongly such as CCL11, CXCL12, and CXCL13, compared with low-affinity binders, such as CCL3 and CCL4, being unable to induce this synergy.

A similar phenomenon was observed for CCL18, an interesting chemokine in that is has been shown to be unregulated in many pathological conditions, yet its receptor remained elusive until it was shown recently to activate CCR8 (48). Moreover, CCL18 is always present at considerably higher concentrations in the circulation than most chemokines, and it was shown to displace certain chemokines bound to heparin (49). This property led to the hypothesis that it could prevent the recruitment of leukocytes by these chemokines by removing them from the endothelial surface.

Since chemokine cooperativity *via* GAG binding would allow chemokines to activate their cognate receptors at lower chemokine concentrations, it is likely that *in vivo*, this phenomenon would extend the range from which chemokines can induce recruitment of leukocytes (50). GAG binding and/or formation of heterocomplexes can definitively contribute to the fine-tuning modulation of chemokine activities occurring *in vivo*.

It is well established that many chemokines exist in equilibrium between the monomeric and dimeric state, and even as higher order oligomers (51–53). It is therefore clear that chemokine biology is more complex than simple monomolecular ligand–receptor interactions. It has been shown *in vitro* that the quaternary structure of chemokines influences the affinity of binding to GAGs (54, 55). Moreover, *in vitro* studies have suggested that dimerization may also occur after binding to GAGs on endothelial cells (56). In fact, this phenomenon is essential for certain chemokines *in vivo* since obligate monomers of the proinflammatory chemokines, CCL2, CCL4, and CCL5, are unable to recruit cells when injected into the peritoneal cavity (44).

It is however important to note that alterations in GAG composition can occur in several pathological conditions (57–59). In addition, chemokine receptors can be found as dimers and/or oligomers at the cell surface (60–62). Due to the complexity of chemokine binding and signaling (63), several mechanisms have been proposed to provide an explanation for synergy between chemokines in leukocyte migration. It is now evident that the synergism between chemokines is crucial at the very early stage of inflammation, as *in vivo* disruption of pro-atherogenic heteromers of CCL5 and CXCL4 resulted in a significant decrease in atherosclerotic lesion formation (38, 64). Moreover, disruption of the heteromers, formed between CCL5 and the α-defensin HNP1, attenuated monocyte and macrophage recruitment in a mouse model of myocardial infarction (65). On the contrary, the study of the role of synergy-inducing chemokines in the tumor microenvironment is at its infancy, as it has been shown *in vitro* that the distinct co-expression of B and T cell attractant chemokines, present in the tumor microenvironment, control cell trafficking of both tumor-infiltrating lymphocytes and malignant B cells (35).

## Chemokines and DAMPs

Under inflammatory conditions, the cross talk between different molecules plays a crucial role in reaching the balance in tissue regeneration. A complete system for the detection, containment, and repair of damage caused to cells in the organism requires warning signals for the cells to respond. These warning signals are called endogenous damage-associated molecular patterns (DAMPs) or alarmins. In addition to the several chemokines that act as enhancers of molecules of the same family, by forming a heterocomplex with chemokine receptor agonists, we have recently identified HMGB1, an alarmin, as an enhancer of the activity of CXCL12 (Figure 1B) (66–68). The heterocomplex HMGB1/CXCL12 can be disrupted with a specific molecule, glycyrrhizin, which inhibits cell influx into the injured tissue. This indicates that a number of components, in addition to the direct activation of the receptor *via* a selective agonist, can regulate chemokine functions *via* a direct interaction with chemokines or chemokine receptors. Multiple chemokines within inflamed tissues selectively enhance each other’s migratory functions, depending on their concentrations, proximity, and simultaneous exposure to leukocytes. The mechanisms underlying the involvement of endogenous DAMPs in chronic diseases are still largely unexplored, and the interaction with other molecules might be a possible approach to understand their targets and functions. The interaction between chemokines and inflammatory molecules needs to be taken into account when chemokine cleavage by proteolysis, or chemokine degradation by atypical chemokine receptors, would be beneficial to achieve a resolving microenvironment favorable for resolution of inflammation by abrogating chemokine signals and the recruitment of inflammatory cells (69). The heterocomplex HMGB1/CXCL12 was demonstrated to prevent CXCL12 degradation (70), similarly to the observation that the complex CCL19/CCL7 prevents CCL7 degradation by the atypical receptor ACKR2 (34).

## Future Perspectives

The chemokine system remains a promising biological target for the development of new therapeutic tools for the treatment of immunological disorders. Nevertheless, drug discovery programs have not yet produced successful drugs targeting the chemokine system for the treatment of inflammatory diseases. Most of the competitive chemokine receptor antagonists developed by all major pharma companies have been disappointingly unsuccessful when tested in clinical trials (71), and as a matter of fact, the only two small molecule inhibitors approved by the FDA do not target inflammation. Taking into account GAGs-binding properties, synergy induced by heterocomplexes formed with non-ligand chemokines or inflammatory molecules, and the possibility that the heterocomplexes might induce differential signaling pathways, will certainly help in elaborating the biology involved in this family and will surely contribute to the successful development of inhibitors of the chemokine system as therapeutics.

## Author Contributions

All authors listed have made substantial, direct, and intellectual contribution to the work and approved it for publication.

## Conflict of Interest Statement

The authors declare that the research was conducted in the absence of any commercial or financial relationships that could be construed as a potential conflict of interest.
